# 

**Published:** 1879-08-20

**Authors:** 


### EDITORIAL AND MISCELLANEOUS.

☞ *The Red Cross* on your Journal shows that you have not paid your subscription. It is notice to subscribers of both 1878 and 1879. Friends, please attend to this matter at once.

---

*The Southern Medical College.*—See the card of the above Institution in the present issue of the Journal.

We assure our readers that this will be an Institution of a high order. The founders are determined to make it so. The college building is rapidly approaching completion, and lectures will open on second Monday in October, as per announcement.

---

*Incubation.*—This word, in medicine, is figuratively applied to the length of time a morbific principle remains undeveloped in the animal economy. There seems to be a law specially governing the period of development in every particular poison—yet exceptions occur in which from some unexplained cause the morbid action is retarded or remains dormant beyond its proper period.

In hydrophobia, the incubation is often prolonged for years. A poisonous germ or molecule being encysted or locked within the cicatrix in such manner as not to be absorbed or developed.

The case reported in last number of our Journal by Dr. L. Johnson would seem to indicate that similar delay of development may occur in the action of the vaccine virus. We are not aware that a similar case is on record.

---

### *EDITORIAL NOTICES.*

*International Medical Congress.*—The International Medical Congress meets in Amsterdam, September 12th, 1879.

*The American Academy of Medicine.*—The objects of the Academy are thus stated in its constitution:

1. To bring those who are alumni of collegiate, scientific and medical schools into closer relations with each other.
2. To encourage young men to pursue regular courses of study in classical or scientific institutions before entering upon the study of medicine.
3. To extend the bounds of medical science, to elevate the profession, to relieve human suffering, and to prevent disease.

The next meeting of this Association will take place in New York, September 16th, 1879.

*OUR ADVERTISING DEPARTMENT.*—This Journal has persistently sought to furnish our readers with advertisements from safe and reliable advertising Houses. We shall continue to do so, and, without encroaching upon our reading matter, which will remain the same, believing that our readers desire it and will be profited thereby.

If it is important to know *what* medicines to prescribe, it it certainly not less important to know *where* and from *whom* to get those that are pure and reliably prepared. We shall hereafter endeavor to keep our readers posted more particularly as to the safe and reliable drug Houses, that they may order from them, or get their home merchants and druggists to do so.

☞ See interesting advertisement of Medical and Scientific Books, Mineral Specimens, etc., by Prof. A. E. Foote, M.D., of Philadelphia.

---

MEDICAL BOOKS.—We ask attention to advertisement of Messrs. Lynch & Thornton, book merchants of Atlanta.

---

RECEIPTED.—1879: W. H. Wilson; Thos. J. Hinsley; H. T. Sheill; E. B. Toungells; C. C. Hart; J. S. Hamilton; T. S. Fox; C. G. Nichols; E. L. Speer; Amos Whidby; James Joyner; R. L. Loamax; T. T. Rodman; K. Smith; J. Hodson; M. Malona; T. T. Farmer; Robert Lake; B. Asbury; S. G. Morley; Wooley B. M.—1878: Joseph Jones; T. B. Lanier.

